# Anisodamine microcirulatory effects in septic shock: be aware of cardiac side effects

**DOI:** 10.1186/s13054-021-03854-5

**Published:** 2021-12-16

**Authors:** Patrick M. Honore, Sebastien Redant, Thierry Preseau, Bogdan Vasile Cismas, Keitiane Kaefer, Leonel Barreto Gutierrez, Sami Anane, Rachid Attou, Andrea Gallerani, David De Bels

**Affiliations:** 1 ICU Dept, Centre Hospitalier Universitaire Brugmann-Brugmann University Hospital, Place Van Gehuchtenplein, 4, 1020 Brussels, Belgium; 2 ED Dept, Centre Hospitalier Universitaire Brugmann, Brussels, Belgium

**Keywords:** Anisodamine, Septic shock, Important cardiac side effects

With great interest, we read the recently published meta-analysis by Yu et al. stating that anisodamine was able to improve microcirculation in patients with septic shock, as supported by lower serum lactate levels and less vasopressor requirements in the treated group [1]. However, the mortality rate in the treated group was lower than that in the usual care group, suggesting that this non-significant finding might be attributable to the limited sample size of the current study [1]. The authors describe the potential beneficial effects of anisodamine in detail, but do not mention the numerous side effects. Anisodamine, a tropane alkaloid extracted from the root of *Anisodus tanguticus* (Maxim.) Pascher in the family Solanaceae, acts as non-specific muscarinic cholinoceptor antagonist competing with acetylcholine for binding to the muscarinic cholinergic receptor, blocking nerve impulses and physiological functions associated with cholinergic neurotransmission [2]. The autonomic receptors distributed in the cardiac, vascular, and airway smooth muscle are mainly muscarinic cholinoceptors [2]. Thus, the antimuscarinic activity of anisodamine can potentially lead to various physiological effects on the cardiorespiratory system [2] and early experimental and clinical studies have indeed reported that structurally-related tropane alkaloids (such as anisodamine, anisodine, scopolamine, and atropine) have potentially undesirable effects on the particularly the cardiovascular systems [2], such as tachycardia and induction of arrhythmia [2] especially atrial arrhythmia [3]. Anisodamine is also know to increase the heart oxygen consumption and can cause torsade de pointes by increasing the QT interval [4]. Anisodamine induces coronary blood vessel dilation, increasing coronary blood flow, while also possibly inducing simultaneous elevation of the ventricular fibrillation threshold [5].

## Data Availability

Not applicable.
