# Supplementary material for: Anisodamine microcirulatory effects in septic shock: be aware of cardiac side effects
Source: Crit Care. 2021 Dec 16;25:433. doi: 10.1186/s13054-021-03854-5 (PMC8680269; doi:10.1186/s13054-021-03854-5)
Supplement: Related texts

# Reply: Authors’ response

**Authors:** Yuetian Yu, Cheng Zhu, Zhongheng Zhang

**Affiliations:** 3 grid.16821.3c 0000 0004 0368 8293 Department of Critical Care Medicine, Ren Ji Hospital, School of Medicine, Shanghai Jiao Tong University, Shanghai, China; 4 grid.16821.3c 0000 0004 0368 8293 Department of Disease Prevention and Control, Rui Jin Hospital, School of Medicine, Shanghai Jiao Tong University, Shanghai, China; 5 grid.16821.3c 0000 0004 0368 8293 Department of Emergency Medicine, Rui Jin Hospital, School of Medicine, Shanghai Jiao Tong University, Shanghai, China; 6 grid.13402.34 0000 0004 1759 700X Department of Emergency Medicine, Sir Run Run Shaw Hospital, Zhejiang University School of Medicine, Hangzhou, 310016 China

We thank Dr. Patrick Honoré and his colleagues for their comments and for the opportunity to further clarify some of the issues raised in our clinical trial [1]. Our study revealed that anisodamine treatment may decrease the serum lactate levels and the need for vasopressors in patients with septic shock. We fully agree that benefits and risks should be carefully weighed. Therefore, we have already pointed out in the protocol that major adverse events related to anisodamine administration (bowel obstruction, urine retention, tachy arrythmias) should be pre-specified in the case report form and should be screened daily by the investigators [6].

Arrhythmia can be noticed easily, which include atrial fibrillation, ventricular tachycardia and even ventricular fibrillation. In a multicenter, randomized trial, it was indicated that arrhythmia was recognized in 207 (24.1%) shock patients with dopamine treatment and 102 (12.4%) shock patients under norepinephrine treatment (*p* < 0.001) [7]. We acknowledge the fact that the antimuscarinic activity of anisodamine can potentially lead to various physiological effects on the cardiorespiratory system, especially atrial arrhythmias [3]. However, it is difficult to ascribe the side effects to anisodamine administration or the vasopressor treatment.

Our study included 355 patients with septic shock, including 22 (6%) with heart failure (11 in each group). Data of these patients was re-analyzed and it showed that atrial fibrillation was recognized in 4 patients in the anisodamine group and 3 patients in the control group. For the entire population, arrhythmias were found in 15 (8.3%) patients in the anisodamine group and 18 (10.3%) patients in the control group (*p* = 0.627) during the treatment, which is lower than in the previous study [7]. The side effects of arrhythmias cannot be ignored due to the anisodamine treatment, especially for those with cardiogenic shock and further study is needed to clarify the causality.
